# XSAnno: a framework for building ortholog models in cross-species transcriptome comparisons

**DOI:** 10.1186/1471-2164-15-343

**Published:** 2014-05-07

**Authors:** Ying Zhu, Mingfeng Li, André MM Sousa, Nenad Šestan

**Affiliations:** Department of Neurobiology, Kavli Institute for Neuroscience, Yale School of Medicine, 06510 New Haven, CT USA; Graduate Program in Areas of Basic and Applied Biology, Abel Salazar Biomedical Sciences Institute, University of Porto, 4099-003 Porto, Portugal

**Keywords:** Comparative transcriptomics, Ortholog annotation, RNA-seq, Gene expression, Prefrontal cortex, Evolution, Human evolution, Primate, Macaque, Chimpanzee

## Abstract

**Background:**

The accurate characterization of RNA transcripts and expression levels across species is critical for understanding transcriptome evolution. As available RNA-seq data accumulate rapidly, there is a great demand for tools that build gene annotations for cross-species RNA-seq analysis. However, prevailing methods of ortholog annotation for RNA-seq analysis between closely-related species do not take inter-species variation in mappability into consideration.

**Results:**

Here we present XSAnno, a computational framework that integrates previous approaches with multiple filters to improve the accuracy of inter-species transcriptome comparisons. The implementation of this approach in comparing RNA-seq data of human, chimpanzee, and rhesus macaque brain transcriptomes has reduced the false discovery of differentially expressed genes, while maintaining a low false negative rate.

**Conclusion:**

The present study demonstrates the utility of the XSAnno pipeline in building ortholog annotations and improving the accuracy of cross-species transcriptome comparisons.

**Electronic supplementary material:**

The online version of this article (doi:10.1186/1471-2164-15-343) contains supplementary material, which is available to authorized users.

## Background

The accurate characterization and quantification of orthologous transcripts across species are critical for understanding the evolution of gene expression and the transcriptome–phenotype relationship. Previous comparative studies have shown that the evolutionary changes in gene expression play a key role in phenotypic changes between species, including the differences between human and closely related non-human primates [1, 2].

The development of sequencing technology, such as RNA-seq, has provided significant advantages over previous microarray technology, for quantifying expression divergence. RNA-seq does not rely on specific predesigned probes or *a priori* knowledge of the transcriptome under investigation, thereby theoretically allowing unbiased whole transcriptome profiling of any species and performing cross-species comparisons [3]. Furthermore, in contrast to microarray, where even a single nucleotide mutation in probe sequence may affect the efficiency of probe hybridization, RNA-seq is more robust to sequence variations between species. However, comparing transcriptomes of different species using RNA-seq is challenging. One critical challenge is the lack of high-quality annotation of orthologous genes. Although multiple databases, such as Ensembl homologs [4], OrthoDB [5] and eggNOG [6], provide a catalog of orthologs between species, none of them provide coordinates of corresponding orthologous regions on reference genomes, which makes it difficult to employ them for RNA-seq analysis. Prevailing annotations for cross-species RNA-seq analysis are based on sequence conservation through either whole genome alignment or local alignment, and have been previously implemented in analyzing transcriptional differences between humans and non-human primates [7–10].

Another challenge in cross-species transcriptome comparisons is the variation of short-read mappablity to orthologs among species. Although the leading short read mapping algorithms all try to identify the best mapping position for each read, a read may still map equally well or nearly equally well to multiple positions because of paralogous sequences in the reference genome [11]. Furthermore, a previous study has shown that mappability varies greatly between species and gene classes [12]. In RNA-seq analysis, the quantification of gene expression will thus be affected by the existence of paralogous sequences. The problem becomes apparent when we perform differential expression analysis between species. A gene may be falsely identified as differentially expressed gene due to differences in mappability between species.

Here, we first analyzed the bias in estimating inter-species difference in expression caused by inter-species difference in mappability based on current annotations, using a published dataset consisting of RNA-seq and high-density exon array. We then created a pipeline named XSAnno, which generated a model of orthologs by combining whole genome alignment, local alignment and multiple filters to remove regions with difference in mappability (DIM) between species. The steps in our computational pipeline are inspired by common practice for annotating orthologous regions, but were modified to suit the specific aim of comparative transcriptome analysis. To assess our method, we performed RNA-seq on dorsolateral prefrontal cortex (DFC) of 5 humans, 5 chimpanzees and 3 rhesus macaques and benchmarked the performance of XSAnno on identifying differentially expressed (DEX) genes between species, by comparing with annotations used in previous studies [7–10]. Validation by ddPCR revealed that our approach greatly reduced the false positives, while keeping the number of false negatives low.

## Results and discussion

### Differences in mappability between species skew gene expression comparisons

To assess the effects of inter-species difference in mappability on estimating inter-species difference in expression using current annotations, we took advantage of a published dataset including RNA-seq and high-density human exon junction array data from cerebellum of human, chimpanzee and rhesus macaque [8]. The RNA-seq data included a total of five lanes of 36 bp single-end reads with two technical replicates for human and macaque and one lane for chimpanzee (Additional file 1: Table S1). The microarray data included 3 replicates of human, chimpanzee and rhesus macaque cerebellum samples (Additional file 1: Table S1). To avoid bias in gene expression quantification, only microarray probes that perfectly matched the genome sequences of all three species were used. As microarray probes were designed to uniquely detect a set of known genes, microarrays are less biased by inter-species differences in mappability than RNA-seq. Therefore, we tested the performance of annotations generated by two most widely used approaches by comparing them with the microarray data. One set of annotation was built based on Ensembl annotation (V64) [4] through whole genome alignment as described in the original study and other studies [7, 9] (WGA annotation, see Methods). The other set was originally built in Blekhman et al. [10] and updated in Primate Orthologous Exon Database (POED), which includes a catalog of unique, non-overlapping, 1:1:1 orthologous exons of human, chimpanzee and rhesus macaque indentified through local alignment from Ensembl annotation.

**Figure 1:**
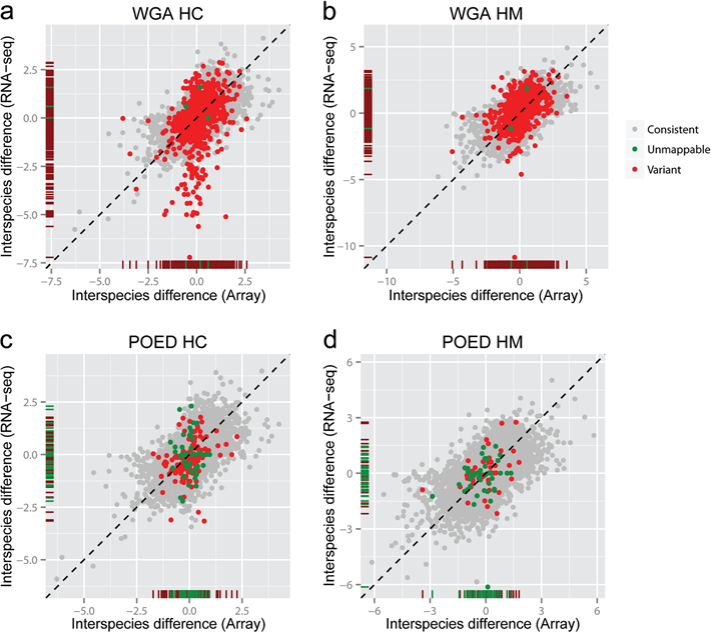
**The effects of different mappabality between species on estimating inter-species gene expression differences.** Inter-species gene expression differences estimated by RNA-seq were plotted against inter-species differences estimated by microarray, using WGA annotation **(a, b)** and POED annotation **(c, d)**. The inter-species differences were calculated as log2 fold change of RPKM (RNA-seq) or intensity (microarray). The rug plots along x and y axes show the distribution of interspecies differences estimated by microarray and RNA-seq, respectively. DIM genes (red) and genes without simulated reads mapped (green) show larger inter-species variation in RNA-seq than in microarray. **(a, c)** Comparison between human and chimpanzee (HC). **(b, d)** Comparison between human and rhesus macaque (HM).

In the WGA annotation, 11,420 human-chimpanzee orthologs and 11,461 human-macaque orthologs were shared with microarray. In POED annotation, 11,266 1:1:1 human-chimpanzee-macaque orthologs were shared with microarray. To identify genes with difference in mappability (DIM genes), we generated ten lanes of simulated RNA-seq (s-RNA-seq) reads per species based on each set of annotation, with the setting that all the transcripts were equally expressed. DIM genes were identified by DESeq [13] with FDR < 0.01, using s-RNA-seq data. We then plotted the inter-species difference estimated by RNA-seq data against inter-species difference estimated by microarray data (Figure 1). DIM genes in WGA annotation showed larger inter-species difference than genes with consistent mappability between species (consistent genes) based on RNA-seq (human-chimpanzee p < 2.2 × 10^−16^, human-macaque p < 2.2 × 10^−16^; see Methods). On the contrary, DIM genes showed similar inter-species difference to consistent genes based on microarray (human-chimpanzee p = 0.90, human-macaque p = 0.94; see Methods; Figure 1a, b). The difference between RNA-seq and microarray suggested that variations in mappability affected the estimation of inter-species difference. As expected, POED annotation included fewer genes with variant mappability between species, because it is a set of orthologs shared by three species and built with local alignment, which is more stringent in terms of sequence conservation, compared with WGA annotation (Figure 1c, d). We observed similar larger inter-species difference of DIM genes estimated by RNA-seq than by microarray using POED annotation (RNA-seq: human-chimpanzee p = 0.005, human-macaque p = 0.09; microarray: human-chimpanzee p = 0.88, human-macaque p = 0.22; see Methods). Besides, more genes with no s-RNA-seq reads aligned were identified using POED annotation, suggesting shortened gene length during the process of ortholog identification (Additional file 2: Figure S1). The inter-species differences of these genes were also more dispersed from 0 in RNA-seq than in microarray (Figure 1), suggesting that the gene expression cannot be well represented if the gene model is truncated too much in the process of ortholog identification.

Another problem with using only local alignment is the loss of syntenic information of genome. In POED annotation, we found some human orthologs in chimpanzee or macaque with exons located in unreasonably distant genomic regions. For example, in POED, the length of *RIN3* is around 130 kb in human, but ~ 125 Mb in macaque, including an 125 Mb intron.

### Outline of the XSAnno framework

**Figure 2:**
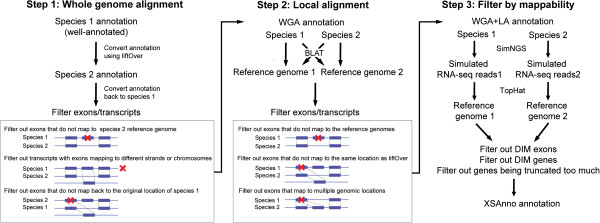
**The XSAnno pipeline.** Pipeline for building ortholog annotation. Blue boxes denote exons and red crosses label the exons or transcripts filtered out.

To fit the aim of RNA-seq analysis, we developed the XSAnno framework for annotating orthologous regions for cross-species gene expression comparisons. XSAnno integrates whole genome alignment, which preserved syntenic information of genome and local alignment, which removes exons that are not highly conserved in sequence with multiple filters, which filters out exons and genes with varied mappability between species (Figure 2):

(i) Our pipeline started with whole-genome alignment (WGA), which preserves syntenic information of the genome. We use UCSC liftOver tool [14], which converts the genome coordinates between species based on whole genome alignment. We select one species (Sp1), usually the one with better annotation, as reference species and lift the annotation to the other species (Sp2). The lifted annotation on Sp2 is then lifted back to the genome of Sp1. The parameters of liftOver are carefully selected by bootstrapping (Supplementary Methods & Additional file 2: Figure S2). In the process, we filter out exons that cannot be lifted from Sp1 to Sp2, exons cannot be lifted back to the original genomic location of Sp1, and transcripts without all exons lifted to the same chromosome or strand.
(ii) We then perform local alignment (LA) to remove exons that are not highly conserved in sequence and exons that may cause ambiguity in RNA-seq read mapping. We align the exons from step one of both species to their reference genome and the reference genome of the other species, respectively, using BLAT [15]. Only exons with a unique conserved ortholog but without highly conserved paralogs are kept. Thresholds of inter- and intra-species percent identity (PID, http://genome.ucsc.edu/FAQ/FAQblat.html) and percentage of mapped length (PL) are chosen to maximize the number of retained exons (Supplementary Methods & Additional file 2: Figure S3).
(iii) Finally, we filter out DIM exons and genes. We generate simulated RNA-seq data using simNGS [16] (http://www.ebi.ac.uk/goldman-srv/simNGS/), incorporating sequencing errors, and setting all transcripts to be equally expressed. With this setting, exons and genes with different mappability of s-RNA-seq reads show statistically significant differential expression and are therefore removed. Besides, we remove genes that are truncated too much (see Methods).

### Generation of human-chimpanzee and human-macaque annotations by XSAnno

As an example of comparisons between closely-related species, our pipeline was first applied to generate human-chimpanzee orthologous genes based on human gene annotation (Ensembl v64) [4], human reference genome (hg19) [17], and chimpanzee reference genome (panTro2) [18]. Starting with 54,127 genes (21,165 protein-coding genes) in Ensembl human gene annotation, we identified 37,662 human-chimpanzee orthologous genes, including 16,774 protein-coding genes (Table 1 & Additional file 1: Table S2). Higher conversion rates were observed for protein-coding genes and lincRNAs, 79.3% and 73.7%, respectively (Additional file 1: Table S2).

**Table 1:** **Number of genes in the annotation**

Species	Annotation Name	Protein coding	Pseudogene	Processed transcript	lincRNA	Others	Total
Human-chimpanzee	WGA	19177	9348	5265	5173	7965	46928
	WGA+LA	18272	6796	4831	4825	6257	40981
	XSAnno	16774	6205	4296	4241	6146	37662
Human-macaque	WGA	18784	6668	4941	4837	6555	41785
	WGA+LA	17344	2532	3926	3947	2477	30226
	XSAnno	15051	2271	2900	2812	1251	24285
Human-chimpanzee-macaque	POED	17105	2528	3756	3697	2842	29928

As expected, the application of XSAnno to human and rhesus macaque, a pair with a more distant evolutionary relationship, identified fewer orthologs. We identified 24,285 human-macaque orthologous genes, including 15,051 protein-coding genes (Table 1 & Additional file 1: Table S2). Compared with human-chimpanzee orthologs, the decrease in human-macaque orthologs mainly occurred in non-protein-coding genes, particularly pseudogenes (Table 1 & Additional file 1: Table S2) due to the existence of highly conserved paralogs.

The XSAnno started with WGA annotation and filtered exons and genes which were not highly conserved in sequence or different in mappability between species. The XSAnno genes were shorter than WGA genes, as expected, but longer than POED genes (Additional file 2: Figure S1).

**Figure 3:**
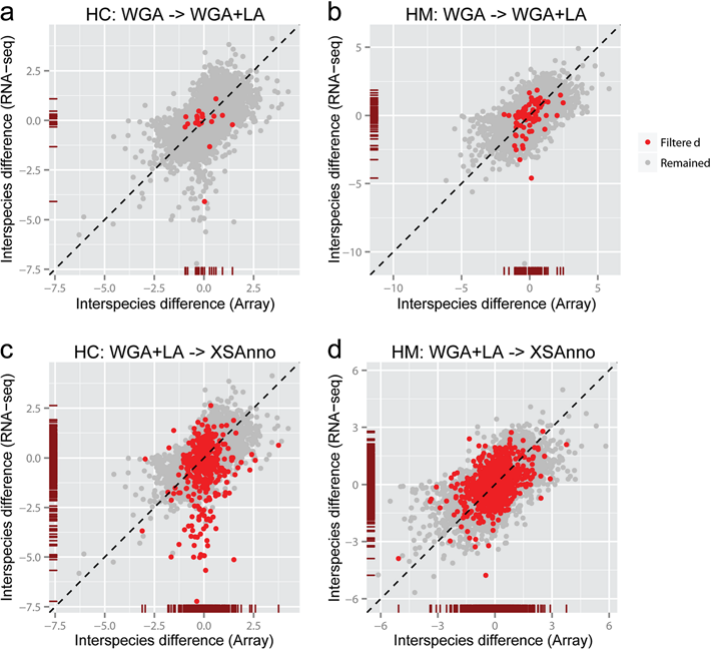
**Assessment of filtering steps in XSAnno using published data.** Comparison of inter-species difference estimated by RNA-seq and inter-species difference estimated by microarray, using WGA annotation **(a, b)** and using WGA+LA annotation **(c, d)**. Genes filtered out in step 2 **(a, b)** and step 3 **(c, d)** are labelled red. These genes display larger inter-species difference using RNA-seq data than using microarray data. **(a, c)** Comparison between human and chimpanzee (HC). **(b, d)** Comparison between human and rhesus macaque (HM).

Each filtering step filtered out genes with large variation in mappability between species (Additional file 2: Figure S4). The genes filtered out displayed larger inter-species variation compared with the remaining genes in RNA-seq (WGA - > WGA + LA: human-chimpanzee p = 6.1 × 10^−3^, human-macaque p = 3.0 × 10^−4^; WGA + LA - > XSAnno: human-chimpanzee p < 2.2 × 10^−16^, human-macaque p = 0.14), but not in microarray (WGA - > WGA + LA: human-chimpanzee p = 0.56, human-macaque p = 0.78; WGA + LA - > XSAnno: human-chimpanzee p =0.09, human-macaque p = 0.82; see Methods; Figure 3), suggesting RNA-seq overestimated inter-species differences of these genes.

### Testing the performance of XSAnno on differential expression analyses

Since the above used published dataset consists of only two technical replicates for human and macaque and no replicates for chimpanzee, it lacks statistical power to identify differentially expressed (DEX) genes. Furthermore, the samples were sequenced as 36 bp single-end reads. Therefore, we performed mRNA-seq (75 bp single-end reads) of DFC tissue from 5 chimpanzee and 3 rhesus macaques (Methods and Additional file 1: Table S3) and compared with the complementary mRNA-seq dataset of 5 human DFC samples generated by the BrainSpan project (http://www.brainspan.org) (Additional file 1: Table S3). The resulting sequencing reads have been deposited to the National Center for Biotechnology Information (NCBI) short-read archive under the accession number PRJNA233428.

The XSAnno human-chimpanzee annotation covered 70.1% chimpanzee RNA-seq reads, which was lower than 77.1% in WGA annotation as expected, but greater than 59.1% in POED (Additional file 2: Figure S5). 90.0% of the human-expressed XSAnno orthologs were also expressed in chimpanzee. Similarly, the XSAnno annotation for human and macaque covered 62.9% macaque RNA-seq reads, greater than 61.6% in POED (Additional file 2: Figure S5). 90.0% of the human-expressed XSAnno orthologs were also expressed in rhesus macaque. Besides, WGA annotation annotates 95.3% and 96.7% junctions identified by TopHat [19] in chimpanzee and macaque, respectively, indicating that the gene structures were preserved in the first step of ortholog identification in our pipeline. The filters applied later reduced the coverage of junctions, but still maintained majority of the junctions, suggesting that our annotation can also be implemented in analyzing alternative splicing (Additional file 2: Figure S6).

To assess the filtering steps in XSAnno pipeline, we first compared the inter-species difference of included genes with that of excluded genes. The genes filtered out in each step showed larger estimated inter-species variation than that of genes remained (p < 2.2 × 10^−16^ in each filtering step in both human-chimpanzee and human-macaque comparisons; see Methods; Additional file 2: Figure S7). To rule out the possibility that our filters selectively removed differentially expressed genes, we compared the inter-species variation of exons from the same gene. Similar to the expression of genes, the expression of retained exons was less variable between species than that of excluded exons from the same gene (p < 2.2 × 10^−16^ in each filtering step in both human-chimpanzee and human-macaque comparisons; see Methods; Additional file 2: Figure S8).

**Figure 4:**
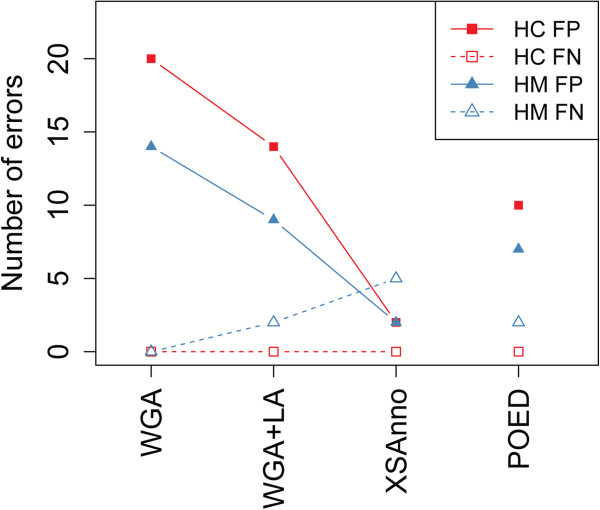
**The performance of XSAnno in inter-species differential gene expression analysis.** Validation by ddPCR. The number of false positives reduced, while the number of false negatives kept low throughout XSAnno filtering steps. HP: comparison between human and chimpanzee; HM: comparison between human and macaque. FP: false positive; FN: false negative.

Since our annotation was designed for cross-species expression comparison, we first assessed the performance of each filtering step in our pipeline. The number of DEX genes was dramatically reduced after filtering (Additional file 2: Figure S9). For validation, we intersected the human-chimpanzee DEX gene list and the human-macaque DEX gene list to differentially expressed in human compared with both chimpanzee and macaque (human DEX genes). The top 10 human DEX genes found only in the WGA annotation, the top 10 human DEX genes in the WGA + LA annotation but not in the XSAnno annotation, and the top 10 human DEX genes in the XSAnno annotation were selected for validation by droplet digital PCR (ddPCR) (Additional file 1: Table S4). As expected, our approach performed better between species with closer evolutionary distance. In the comparison between human and chimpanzee, the number of false positives reduced from 20 using WGA annotation to 2 using XSAnno annotation, while the number of false negatives remained at 0 (Figure 4 & Additional file 1: Table S5). In the comparison between human and macaque, the number of false positives reduced from 14 using WGA annotation to 2 using XSAnno annotation, while the number of false negatives rose to 5 (Figure 4 & Additional file 1: Table S5). Sequence analysis of the genes identified as human DEX only in WGA or WGA + LA annotation revealed the existence of highly conserved paralogs in one species but not in the other, which explained the difference in mappability between species (Additional file 1: Table S6). Among the genes we validated, our pipeline reduced the false positives and kept the false negative rate low, compared with WGA and POED annotations (Figure 4).

## Conclusions

We described a pipeline to build ortholog annotations for comparative transcriptome analysis between closely-related species. The XSAnno pipeline incorporates previous whole genome alignment and local alignment methods with multiple filters to eliminate false positives caused by differences in mappability. Even though our pipeline was tested on human and non-human primate brain transcriptome data, it is not limited to these species.

Our pipeline aims to generate annotation of a conservative set of orthologs to avoid false positives in cross-species analysis. Therefore, it excludes genes with high rate of DNA changes and genes with highly conserved paralogs. Although the level of conservation can be adjusted by tuning parameters in the pipeline to meet specific requirements of each study, separate approaches would be necessary to study genes with large structure changes and duplicated genes.

Compared to existing ortholog databases, the XSAnno pipeline provides a more flexible way to identify orthologs between any pair of closely-related species. It generates gene models that are specifically designed for comparative transcriptome analysis. RNA-seq and ddPCR validation suggest that our approach reduced false positives in cross-species expression analysis, while keeping the false negative rate low. The XSAnno package and pre-processed ortholog annotations of selected species are available in Additional file 3 and can be downloaded at http://medicine.yale.edu/lab/sestan/resources/index.aspx.

## Methods

### Building ortholog annotations

Human-chimpanzee orthologs and human-macaque orthologs were generated separately, based on human Ensembl annotation (v64) [4], human genome (hg19) [17], chimpanzee genome (panTro2) [18] and macaque genome (rheMac2) [20]. The pair-wise alignment files were downloaded from UCSC genome browser (http://genome.ucsc.edu/). Gene annotation of chimpanzee and macaque used for comparison were also obtained from Ensembl (v64) database (http://www.ensembl.org).

### WGA annotation

To keep syntenic information, human exons from all transcripts were lifted to genomic locations on reference genome of chimpanzee and macaque by liftOver tool [14], using pair-wise alignment files downloaded from UCSC genome browser. The liftOver parameter “-minMatch” was set to 0.98 for chimpanzee and 0.913 for macaque, based on bootstrapping (Supplementary Methods & Additional file 2: Figure S2). The lifted exons on reference genome of chimpanzee and macaque were then mapped back to human reference genome, using liftOver tool. During the reciprocal mapping, the following exons/transcripts were excluded: (i) exons cannot be lifted from human to the other species were filtered out; (ii) exons cannot be lifted back to the original genomic location of human genome were filtered out; (iii) transcripts with exons mapped to different chromosomes or strands were filtered out. The process can be completed in one step by running *AnnoConvert* in our pipeline.

### POED annotation

The orthologous exons of human, chimpanzee and macaque were downloaded from Primate Orthologous Exon Database (POED, Version 2; http://giladlab.uchicago.edu/orthoExon/). To be consistent with other databases, we converted genomic coordinates on chimpanzee genome panTro3 to panTro2 by liftOver.

### XSAnno annotation

Step1: The first step is the same as how we build WGA annotation.

Step2: Exons from WGA annotation were aligned to the reference genomes of both the same and the other species by BLAT [15]. Percent identity (PID) and percentage of aligned length (PL) were calculated as measures of local alignment. The thresholds of inter-species and intra-species PID and PL were chosen separately to maximize the number of exons retained (Supplementary Methods and Additional file 2: Figure S3). The inter-species PID and PL were selected to filter out exons without unique, highly conserved orthologs. For human and chimpanzee, the inter-species PID and PL were both set to 0.95. For human and macaque, the inter-species PID and PL were both set to 0.9. Exons that were not aligned to the same genomic location as WGA annotation or were aligned to multiple genomic locations using current cutoff were removed. The intra-species PID and PL were selected to filter out exons with highly conserved regions, which may cause ambiguity in mapping. For both chimpanzee and macaque, the intra-species PID was set to 0.97 and the intra-species PL was set to 0.95. Exons that were aligned to multiple genomic locations of their own reference genome at current cutoff were filtered out. The process can be finished by running *BlatFilter* combined with R [21] functions of threshold determination and filtering.

Step3: To eliminate exons and genes with large inter-species difference in mappability, we generated simulated RNA-seq data with the setting that all transcripts are equally expressed, using simNGS. To run simNGS in parallel with Step Two, we generated simulated HiSeq 100 bp single-end reads based on WGA annotation and then calculated expression only for exons in WGA + LA annotation. Coverage of all transcripts was set to 10X. Ten simulated RNA-seq fastq files were generated for each species. The simulated reads were then mapped to their own genome, using TopHat [19] without providing junction annotation. The number of reads mapped to each exon was counted and used for differential expression analysis with DESeq package [13] for R. Exons and genes that are significantly different between species (FDR < 0.01) were filtered out. Besides, we filtered out genes with length smaller than one third of original length and shorter than 1 kb.

The example scripts to generate simulated reads and to filter exons and genes are available in our pipeline.

### Analysis of published data

Affymetrix Human Exon Junction array data were downloaded from GSE15665. Gene expression was estimated using probes perfectly conserved in nonhuman primates and normalized by quantile normalization as described in the original study.

RNA-seq data were downloaded from SRA023554.1. RNAs were sequenced as 36 bp (human) and 35 bp (chimpanzee and macaque) single-end reads by Illumina GAII. Reads were aligned by TopHat, allowing 2 mismathes, without providing transcriptome annotation. Read count and RPKM of genes were calculated by RSEQTools [22].

Ten lanes of simulated RNA-seq data per species were generated by simNGS, using different sets of annotations. DIM genes were identified by DESeq with FDR < 0.01.

To compare inter-species differences of DIM genes with that of genes with consistent cross-species mappability, we performed the F test for equality of variances. In detail, if mappability affects estimation of inter-species differences, we expect larger variance in inter-species differences of DIM genes than in inter-species differences of consistent genes.  (  and  represent the sample variances of inter-species differences of DIM genes and consistent genes, respectively). The F test was conducted using R function var.test, with alternative hypothesis .

### RNA sequencing and data analysis

#### RNA extraction

Postmortem human brain specimens were obtained from tissue collections at the Department of Neurobiology at Yale University School of Medicine and the Clinical Brain Disorders Branch of the National Institute of Mental Health. Tissue was collected after obtaining parental or next of kin consent and with approval by the institutional review boards at the Yale University School of Medicine, the National Institutes of Health, and at each institution from which tissue specimens were obtained. Tissue was handled in accordance with ethical guidelines and regulations for the research use of human brain tissue set forth by the NIH (http://bioethics.od.nih.gov/humantissue.html) and the WMA Declaration of Helsinki (http://www.wma.net/en/30publications/10policies/b3/index.html). Appropriate informed consent was obtained and all available non-identifying information was recorded for each specimen. Specimens range in age from 21 to 40 years. The postmortem interval (PMI) was defined as hours between time of death and time when tissue samples were frozen.

All experiments using nonhuman primates were carried out in accordance with a protocol approved by Yale University’s Committee on Animal Research and NIH guidelines.

DFC tissue samples were dissected from postmortem adult chimpanzee and macaque brains using the criteria previously described [23, 24]. Human DFC RNA-seq data were generated as a part of the BrainSpan project (http://www.brainspan.org). Together, the RNA-seq dataset includes DFC samples from 5 humans, 5 chimpanzees, and 3 macaques. A bead mill homogenizer (Bullet Blender, Next Advance) was used to lyse the pulverized DFC tissue samples. Each pulverized tissue sample was transferred to a chilled safe-lock microcentrifuge tube (Eppendorf). A mass of chilled stainless steel beads (Next Advance, cat# SSB14B) equal to the mass of the tissue was added to the tube. Two volumes of lysis buffer were added to the tissue and beads. Samples were mixed in the Bullet Blender for 1 min at a speed of six. Samples were visually inspected to confirm desired homogenization and then incubated at 37°C for 5 min. The lysis buffer was added up to 0.6 ml, and samples were mixed in the Bullet Blender for 1 min. Total RNA was extracted using RNeasy Plus Mini Kit (Qiagen) for mRNA-sequencing. Each sample was subjected to a DNase treatment (TURBO DNase, Ambion) as per manufacturers’ instructions.

Optical density values of extracted RNA were measured using NanoDrop (Thermo Scientific) to confirm an A260:A280 ratio above 1.9. RIN was determined for each sample using Bioanalyzer RNA 6000 Nano Kit (Agilent), depending upon the total amount of RNA.

### Library preparation for mRNA-sequencing

cDNA libraries were prepared using the mRNA-Seq Sample Kit (Illumina) as per the manufacturer’s instructions with some modifications. Briefly, polyA RNA was purified from 1 to 5 μg of total RNA using (dT) beads. Quaint-IT RiboGreen RNA Assay Kit (Invitrogen) was used to quantitate purified mRNA with the NanoDrop 3300. Following mRNA quantitation, 2.5 μl spike-in master mixes, containing five different types of RNA molecules at varying amount (2.5 × 10^−7^ to 2.5 × 10^−14^ mol), were added per 100 ng of mRNA [25]. The spike-in RNAs were synthesized by External RNA Control Consortium (ERCC) consortium by *in vitro* transcription of *de novo* DNA sequences or of DNA derived from the *B. subtilis* or the deep-sea vent microbe *M. jannaschii* genomes and were a generous gift of Mark Salit at the National Institute of Standards and Technology (NIST). These were used both to track the brain regions, species and to normalize expression levels across experiments. Each sample was tagged by adding a pair of spike-in RNAs unique to the region from which the sample was taken. Also, an additional three common spike-ins were added for controlling sequencing error rates, which is not influenced by SNP existence (Additional file 1: Table S7). Spike-in sequences are available at http://archive.gersteinlab.org/proj/brainseq/spike_in/spike_in.fa. The mixture of mRNA and spike-in RNAs were subjected to fragmentation, reverse transcription, end repair, 3′– end adenylation, and adapter ligation to generate libraries of short cDNA molecules. The libraries were size selected at 200 – 250 bp by gel excision, followed by PCR amplification and column purification. The final product was assessed for its size distribution and concentration using Bioanalyzer DNA 1000 Kit.

### Sequencing

We used Illumina’s Genome Analyzer IIx (GAIIx) for mRNA-sequencing by loading one sample per lane. For mRNA-sequencing, the library was diluted to 10 nM in EB buffer and then denatured using the Illumina protocol. The denatured libraries were diluted to 12 pM, followed by cluster generation on a single-end Genome Analyzer IIx (GAIIx) flow cell (v4) using an Illumina cBOT, according to the manufacturer's instructions. The Illumina GAIIx flow cell was run for 75 cycles using a single-read recipe (v4 sequencing kits) according to the manufacturer’s instructions.

### Mapping of mRNA-seq reads

We chose TopHat to map RNA-seq reads due to its ability to map junction reads without depending on annotation. The reference genomes used were the same as those for ortholog identification. Only uniquely mapped reads with at most 2 mismatches were included to calculate exon/gene read number and reads per kilobase per million (RPKM) [26].

### Testing the effects of filters

To test the effects of each filtering step, we first compared the inter-species variation of genes remained with the ones filtered out in each filtering step. The inter-species log2-fold-change (log 2FC = log 2(RPKM_sp1_ + 1) − log 2(RPKM_sp2_ + 1)); sp1 and sp2 stand for Species 1 and Species 2, respectively) were calculated for each gene, using WGA annotation, WGA+LA annotation, and XSAnno annotation, respectively. To test the effects of local alignment, we compared the distribution of inter-species log2FC of genes remained in WGA+LA annotation from WGA annotation with that of genes excluded in WGA+LA annotation. Similarly, the distribution of inter-species log2FC of genes remained in XSAnno annotation was compared with the distribution of genes filtered out in XSAnno annotation from WGA+LA annotation. We conducted the F test for equality of variances as used in analyzing the published dataset.

To compare the inter-species variation of included exons with excluded exons from the same transcripts, we summarized the inter-species variation of in and out exons by calculating the mean inter-species log2FC. In other words, for a specific gene, exonFC_in_ = mean (log2FC_in_); exonFC_out_ = mean (log2FC_out_). For each gene, the difference between exons included and excluded was then calculated as In-Out = |exonFC_in_| - |exonFC_out_|. We then performed the paired Wilcoxon signed-rank test with alternative hypothesis |exonFC_in_| < |exonFC_out_| to test whether inter-species difference of in-exons are smaller than that of out-exons.

### Differential expression analysis

Differential expression analysis were performed between human and chimpanzee and between human and macaque, respectively, with DESeq [13] package for R. Genes were identified as DEX, if FDR < 0.01.

The list of human-chimpanzee DEX genes were then intersected with the list of human-macaque DEX genes. Genes with the same direction of change (up or down) in human comparing with other two species were selected as human DEX genes.

### Validation by droplet digital PCR

Thirty genes in the human DEX gene list were selected for validation, including 10 most significant human DEX genes only in WGA annotation, 10 most significant human DEX genes in WGA+LA annotation but not in XSAnno annotation, and 10 most significant human DEX genes in XSAnno annotation (Table S4).

We employed droplet digital PCR (ddPCR) to reliably quantify gene expression. An aliquot of the total RNA that was previously extracted from 3 randomly selected brains per species was used for secondary validation through ddPCR analysis. One μg of total RNA was used for cDNA synthesis using SuperScript III First-strand synthesis Supermix (Invitrogen) and subsequently diluted with nuclease-free water. Custom gene-specific primers and probe for each gene of interest were designed using NCBI/Primer-BLAST (http://www.ncbi.nlm.nih.gov/tools/primer-blast/) and PrimerQuest tool (IDT). In detail, primer pairs were designed in genomic regions that are orthologous (or identical, if the gene is conserved highly across three species), as well as to be separated by at least one intron on the corresponding genomics DNA with a targeted amplicon size at 70 bp to 200 bp. We also allowed primers to amplify mRNA splice variants that are annotated in RefSeq, while did not allow them to contain known SNPs. The probe was designed by PrimerQuest tool (IDT) by applying the above pre-designed PCR primers. We opted to design identical probe sequence for each species, but if the target region is less conserved across three species, we had to design slightly different probes for each species. IDT’s proprietary ZEN internal quencher was applied on top of a 3′ quencher (IBFQ) and a 5′ fluorophore (FAM or HEX) probe labeling. ddPCR was carried out using the Bio-Rad QX100 system. After each PCR reaction mixture, consisting of ddPCR master mix and custom primers/probe set, was partitioned into 15,000–20,000 droplets, parallel PCR amplification was carried out. Endpoint PCR signals were quantified and Poisson statistics was applied to yield target copy number quantification of the sample. Two color PCR reaction was utilized for the normalization of gene expression by the housekeeping gene *TBP*. Table S8 in Additional file 1 provides sequences of primers and probes used for the validation.

Gene expression was calculated as the ratio of target genes to the housekeeping gene *TBP*. Wilcoxon signed-rank tests were performed to identify differentially expressed genes between human and chimpanzee and between human and macaque, separately. Genes were considered as DEX if p ≤ 0.1.

## Electronic supplementary material

Additional file 1: **Table S1.** Sample information of published data. **Table S2.** Gene number in different annotations. **Table S3.** Sample Information of our RNA-seq data. **Table S4.** List of genes for validation. **Table S5.** RNA-seq and ddPCR results of genes for validation. **Table S6.** Paralogs of genes selected for validation. **Table S7.** Spike-in RNAs. **Table S8** List of PCR primers and probes used for ddPCR validation. (XLSX 49 KB)

Additional file 2: **Methods: Determination of liftOver parameters and Determination of BLAT parameters.** **Figure S1.** Distribution of gene length. **Figure S2.** Determination of liftOver parameters. **Figure S3.** Determination of BLAT parameters. **Figure S4.** Distribution of inter-species difference in mappability. Figure S5 Percentage of reads covered by different annotations. **Figure S6.** Percentage of junction reads covered by different annotations. **Figure S7.** The performance of filters on estimating inter-species differences of genes. **Figure S8.** The performance of filters on estimating inter-species differences of exons. **Figure S9.** The number of differentially expressed genes. (DOCX 3 MB)

Additional file 3: **XSAnno pipeline.** (ZIP 21 KB)
