# Supplementary material for: XSAnno: a framework for building ortholog models in cross-species transcriptome comparisons
Source: BMC Genomics. 2014 May 7;15(1):343. doi: 10.1186/1471-2164-15-343 (PMC4035071; doi:10.1186/1471-2164-15-343)
Supplement: Supplementary file 2 — Additional file 2: Methods: Determination of liftOver parameters and Determination of BLAT parameters. Figure S1. Distribution of gene length. Figure S2. Determination of liftOver parameters. Figure S3. Determination of BLAT parameters. Figure S4. Distribution of inter-species difference in mappability. Figure S5 Percentage of reads covered by different annotations. Figure S6. Percentage of junction reads covered by different annotations. Figure S7. The performance of filters on estimating inter-species differences of genes. Figure S8. The performance of filters on estimating inter-species differences of exons. Figure S9. The number of differentially expressed genes. (DOCX 3 MB) [file 12864_2014_6047_MOESM2_ESM.docx]

**Additional file 2 to**

### XSAnno: A framework for building ortholog models in cross-species transcriptome comparisons

**Ying Zhu^1^, Mingfeng Li^1^, André M. M. Sousa^1,2^, Nenad Šestan^1*^**

^1^Department of Neurobiology and Kavli Institute for Neuroscience, Yale School of Medicine,

New Haven, Connecticut 06510, USA

^2^Graduate Program in Areas of Basic and Applied Biology, Abel Salazar Biomedical Sciences

Institute, University of Porto, 4099-003 Porto, Portugal

*To whom correspondence should be addressed. E-mail: [nenad.sestan@yale.edu](mailto:nenad.sestan@yale.edu)

**Table of Contents**

1. **Supplementary Methods**
   1. Determination of liftOver parameters
   2. Determination of BLAT parameters
2. **Supplementary Figures**
3. Distribution of gene length
4. Determination of liftOver parameters
5. Determination of BLAT parameters
6. Distribution of inter-species difference in mappability
7. Percentage of reads covered by different annotations
8. Percentage of junction reads covered by different annotations
9. The performance of filters on estimating inter-species differences of genes
10. The performance of filters on estimating inter-species differences of exons
11. The number of differentially expressed genes
12. **Supplementary Tables (Additional file 1)**
13. Sample information of published data
14. Gene numbers in different annotations
15. Sample Information of our RNA-seq data
16. List of genes for validation
17. RNA-seq and ddPCR results of genes for validation
18. Paralogs of genes selected for validation
19. Spike-in RNAs
20. List of PCR primers and probes used for ddPCR validation
21. **XSAnno pipeline (Additional file 3)**
22. **Supplementary Methods**

**Determination of liftOver parameters**

The parameter “-minMatch” was determined by bootstrapping. In each iteration, a sequence of 150bp (the mean size of an exon) was sampled from each block in the whole genome pairwise alignment file downloaded from UCSC genome browser. The median of the percentage of sequence identity (MPID) of all the sampled short sequences were calculated. We repeated the process for 1000 times. The “–minMatch” is set to the median of MPID from all iterations. Perl script is available in XSAnno package.

**Determination of BLAT parameters**

Percent identity (PID) and the percentage of length aligned (PL) were calculated as measures of local alignment. PID is calculated according to UCSC genome browser percent identity score calculation (<http://genome.ucsc.edu/FAQ/FAQblat.html#blat4>). PL is defined as the percentage of query sequence aligned. For example, for an exon, PL = aligned length / exon length.

The cutoff of PID (PID_c_) and PL (PL_c_) were selected for intra-species alignment and inter-species alignment, separately, to maximize the number of exons retained. The exon passes filter if

1. the exon aligns to a genomic region with PID > PID_c_ and PL > PL_c_ ;
2. only an unique genomic region was identified at current cutoff;
3. the unique target region matches the original location of the exon (for intra-species alignement) or the corresponding location identified by liftOver (for inter-species alignment).

The R functions for threshold selection and exon filtering are in XSAnno package.

1. **Supplementary Figures**

**
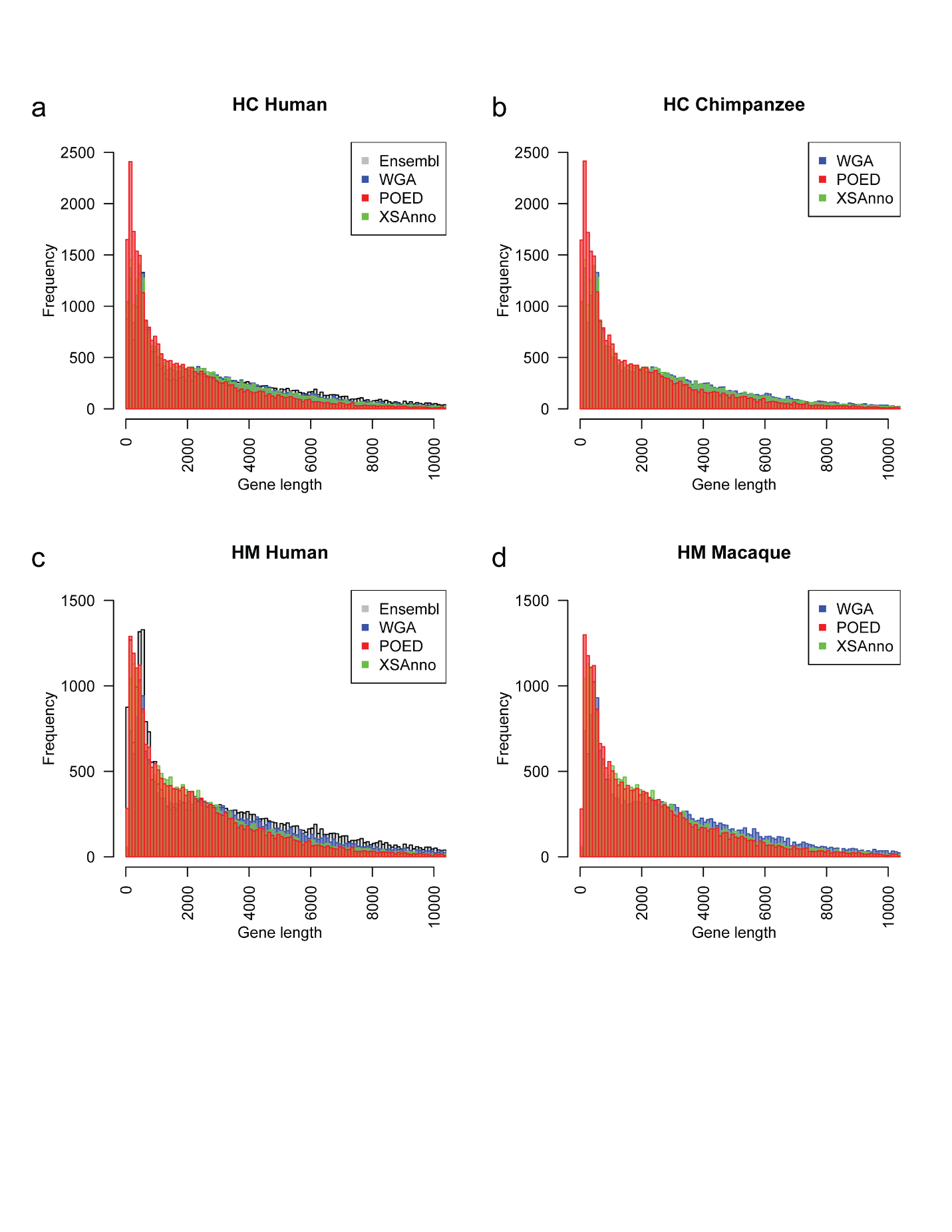
**

**Figure S1. Distribution of gene length**

Comparison of gene length across Ensembl human annotation, WGA annotation, POED annotation and XSAnno annotation, using shared genes. WGA annotation preserved longest gene length, and XSAnno had genes longer than POED annotation. HC: Comparison between human and chimpanzee; HM: Comparison between human and macaque.


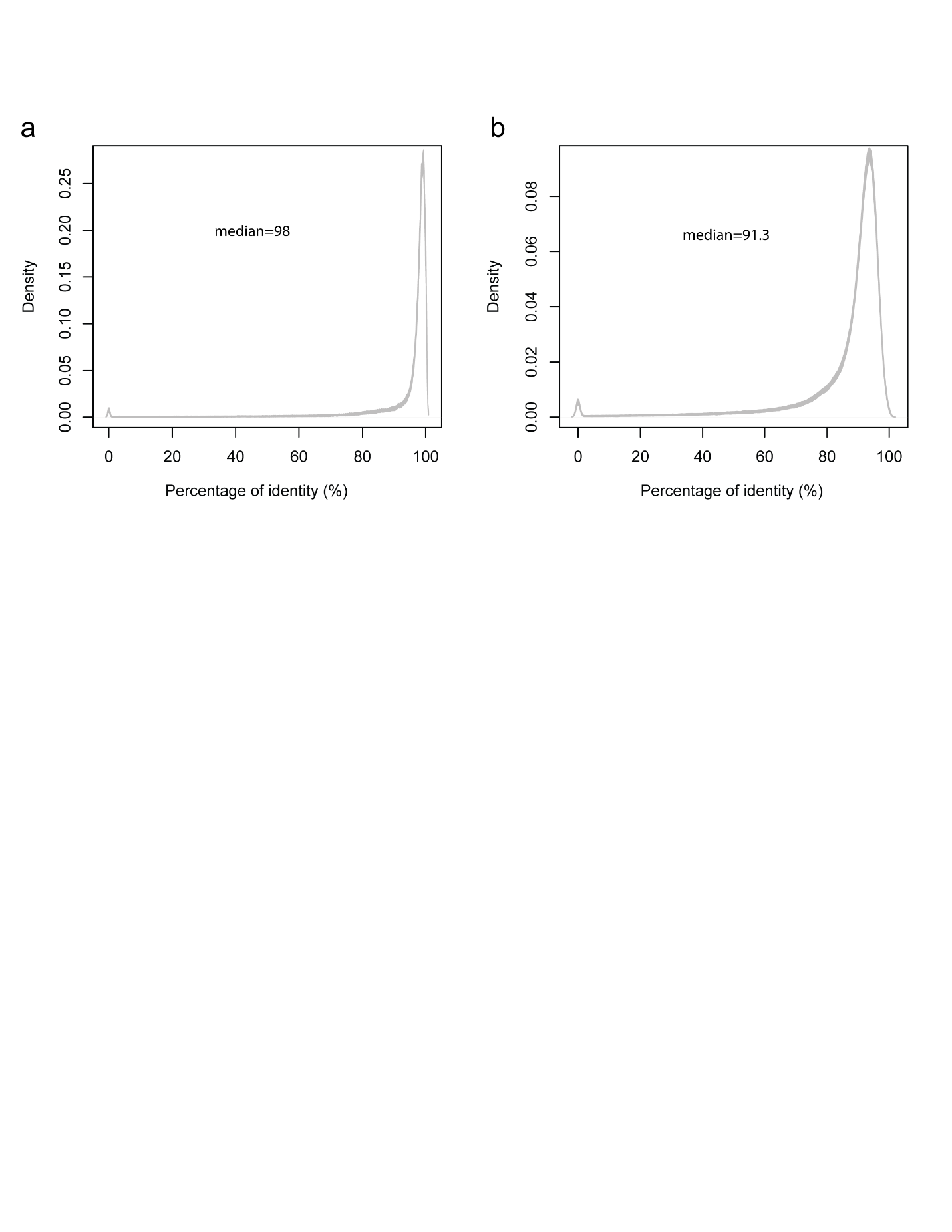


**Figure S2. Determination of liftOver parameters**

The “-minMatch” of liftOver was set to the median value of MPID from 1000 iterations, when generating human-chimpanzee (a) and human-macaque (b) orthologs, respectively.


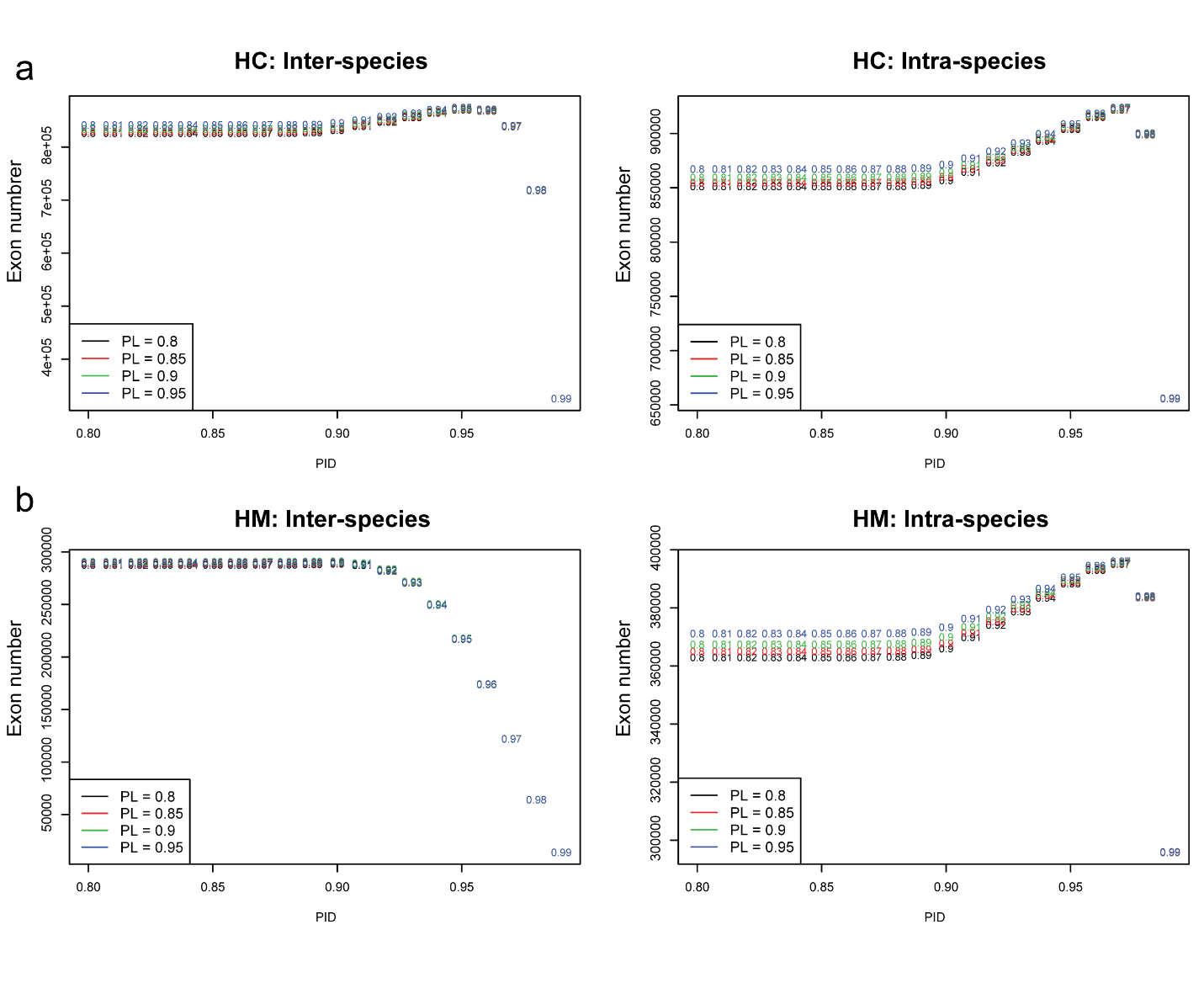


**Figure S3. Determination of BLAT parameters**

Thresholds of inter-species PID and PL (left panels) and intra-species PID and PL (right panels) were selected for human-chimpanzee (a) and human-macaque (b) separately to maximize the number of exons remained in the annotation.


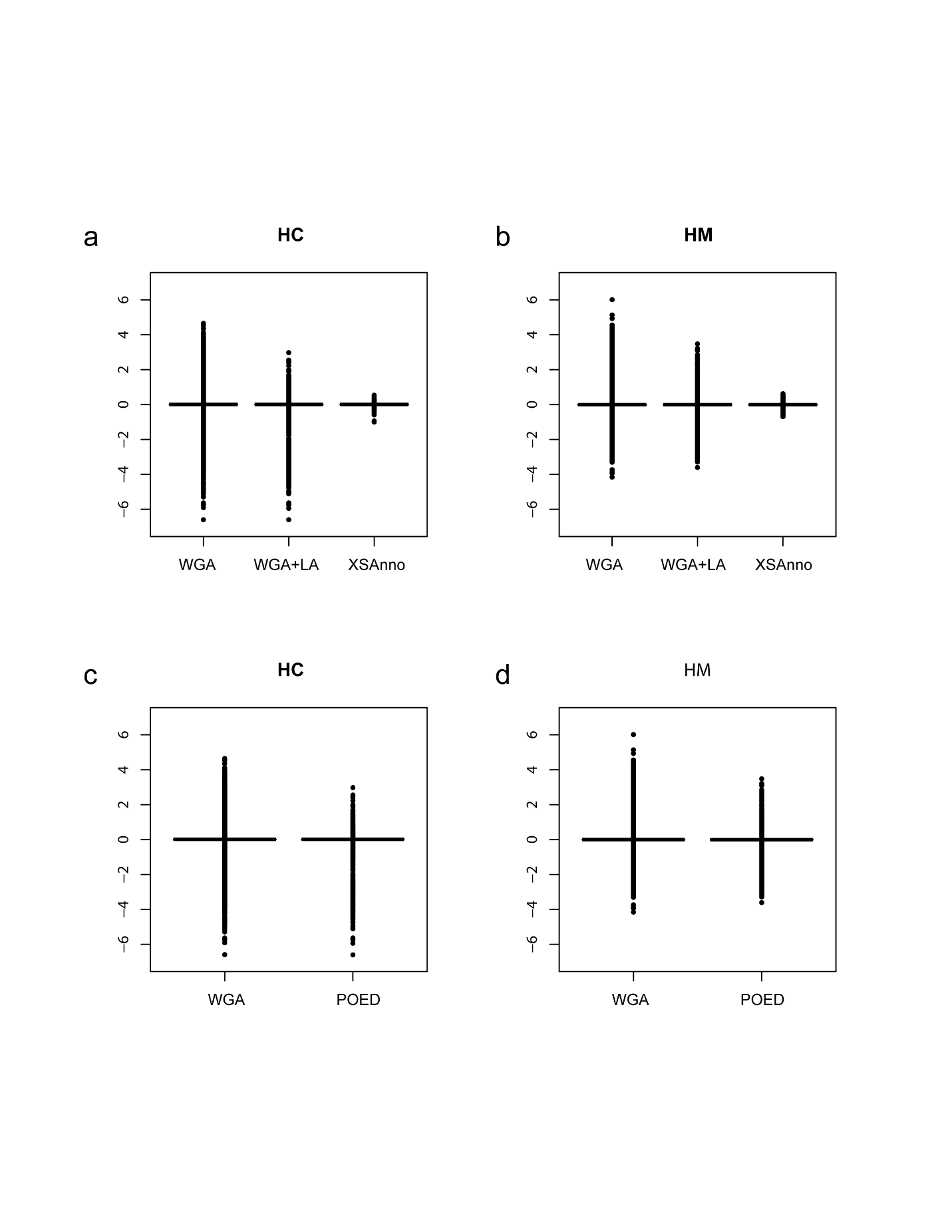


**Figure S4. Distribution of inter-species difference in mappability**

(a, b) Genes with high variation in mappability between species were filtered out in the filtering steps in XSAnno pipeline. (c, d) XSAnno annotation included genes with lower inter-species mappability variation, compared with WGA and POED annotations. HP: human-chimpanzee; HM: human-macaque.


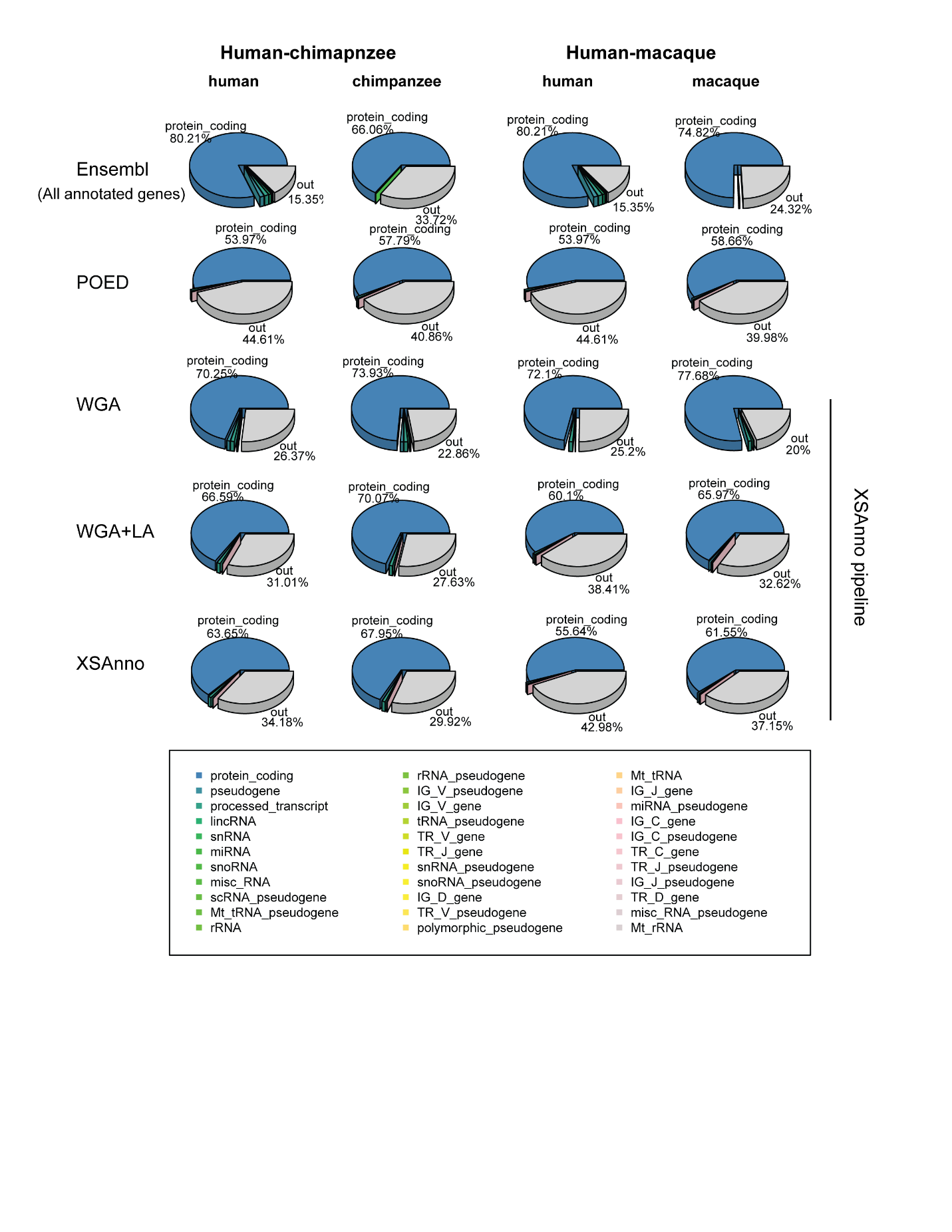


**Figure S5. Percentage of reads covered by different annotations**

The percentage of uniquely mapped reads is presented in the pie charts, using different sets of annotations. From left to right, the columns demonstrate the uniquely mapped human (Column 1) and chimpanzee reads (Column 2) using human-chimpanzee annotations (left two columns) and the uniquely mapped human (Column 3) and macaque reads (Column 4) using human-macaque annotations (right two columns), respectively.

**
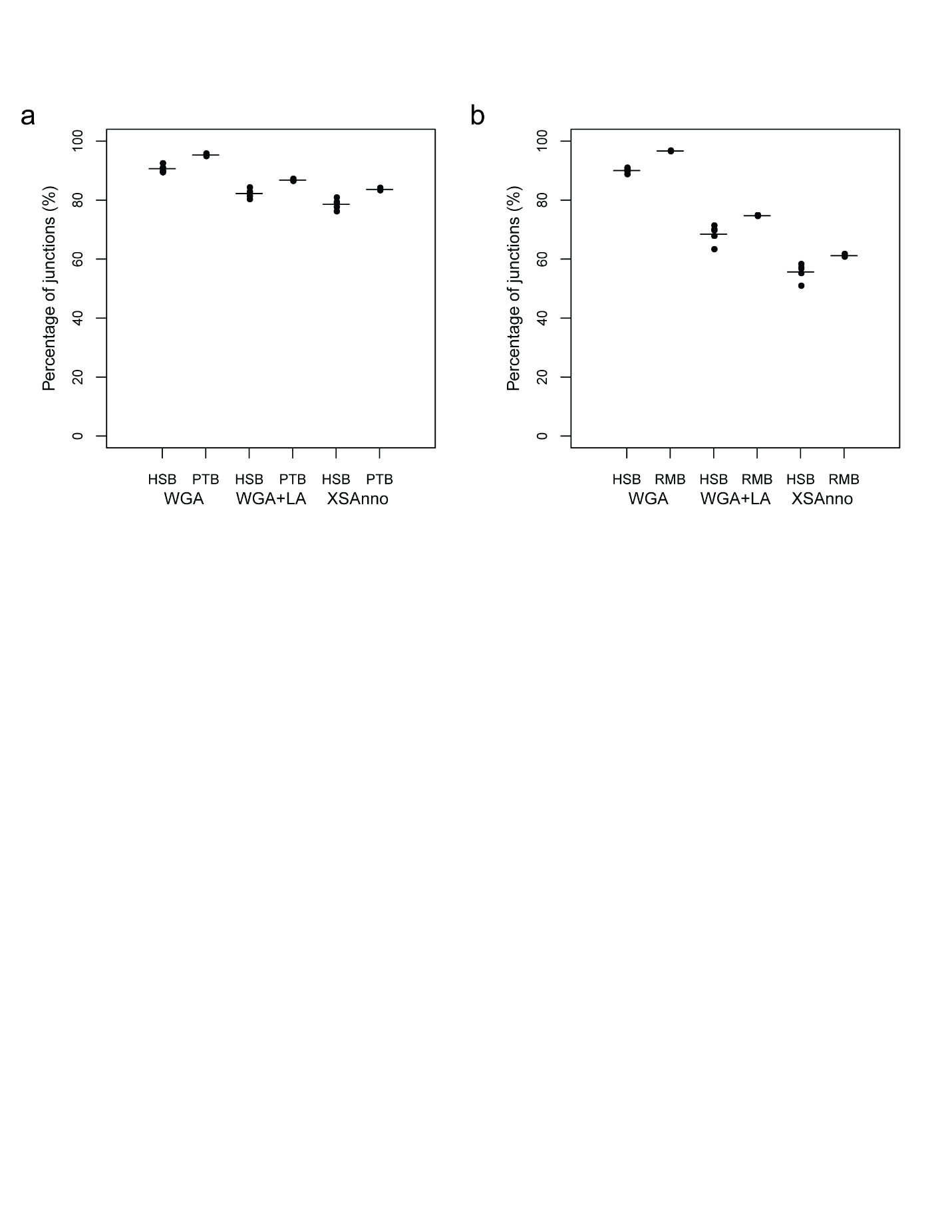
**

**Figure S6. Percentage of junction reads covered by different annotations**

Junction reads were identified by topHat, without providing a reference annotation. (a) Human-chimpanzee. (b) Human-macaque.

**
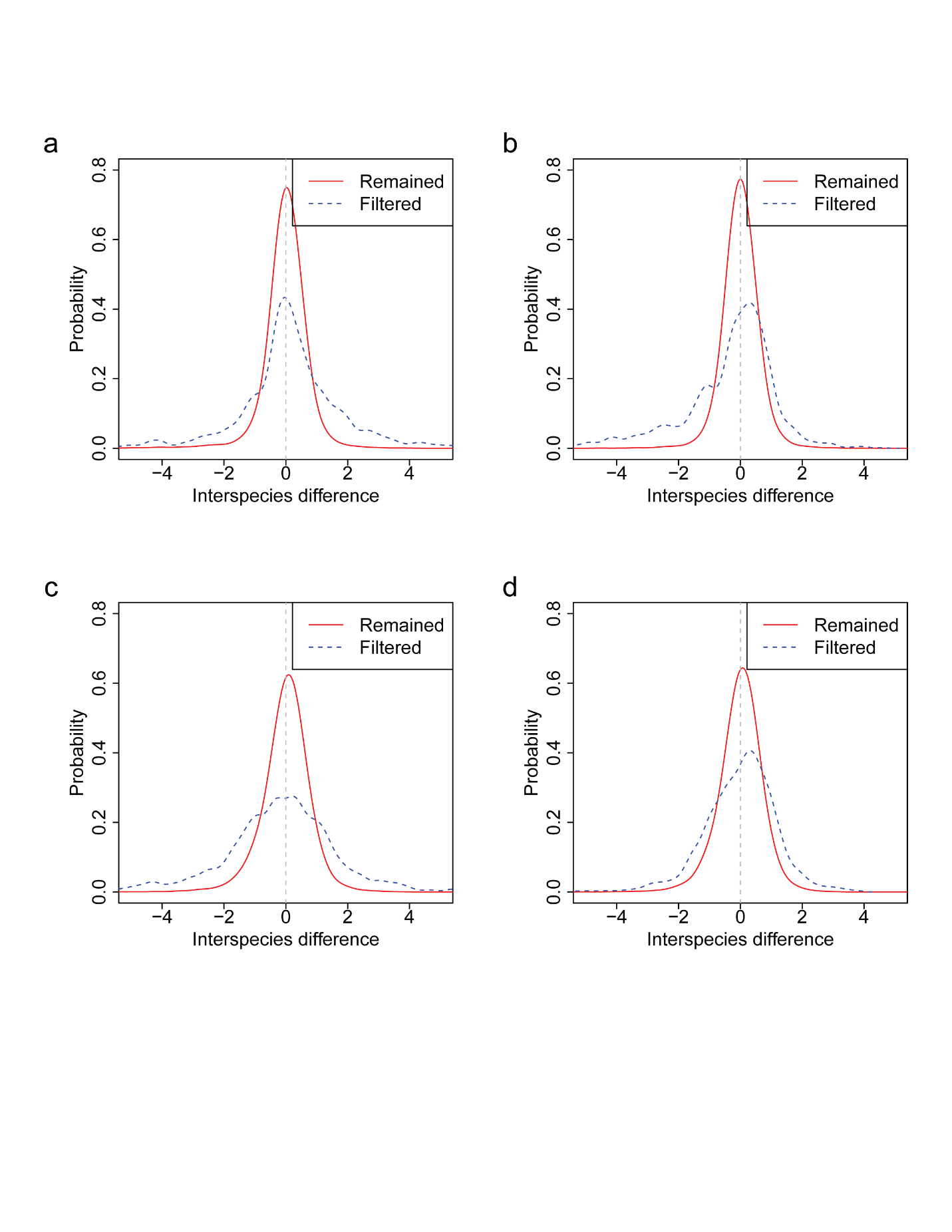
**

**Figure S7. The performance of filters on estimating inter-species differences of genes**

Distribution of inter-species variation of retained (red) and filtered (blue) genes, in each filtering step. Interspecies difference = log2(RPKM_chimp or macaque_ + 1) – log2(RPKM_human_ + 1). (a, c) Comparisons between WGA annotation and WGA+LA annotation. (b, d) Comparisons between WGA+LA annotation and XSAnno annotation. (a, b) Comparisons between human and chimpanzee. (c, d) Comparisons between human and macaque.


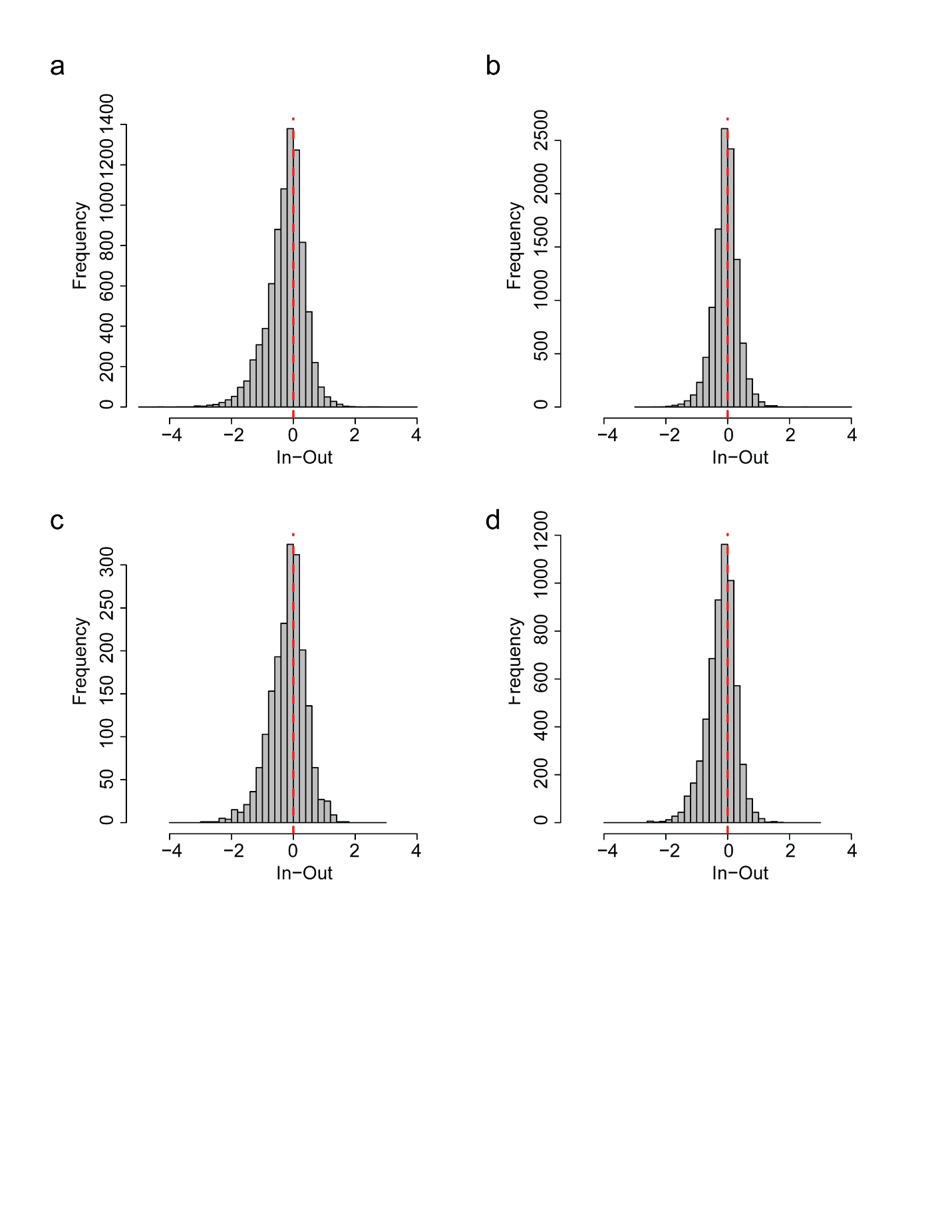


**Figure S8. The performance of filters on estimating inter-species differences of exons**

Distribution of inter-species differences between included (In) and excluded (Out) exons from the same transcript in inter-species difference. Interspecies difference = log2(RPKM_chimp or macaque_ + 1) – log2(RPKM_human_ + 1); In-Out = mean(Interspecies difference_In_) – mean(Interspecies difference_out_). (a, c) Comparisons between WGA annotation and WGA+LA annotation. (b, d) Comparisons between WGA+LA annotation and XSAnno annotation. (a, b) Comparisons between human and chimpanzee. (c, d) Comparisons between human and macaque.

**
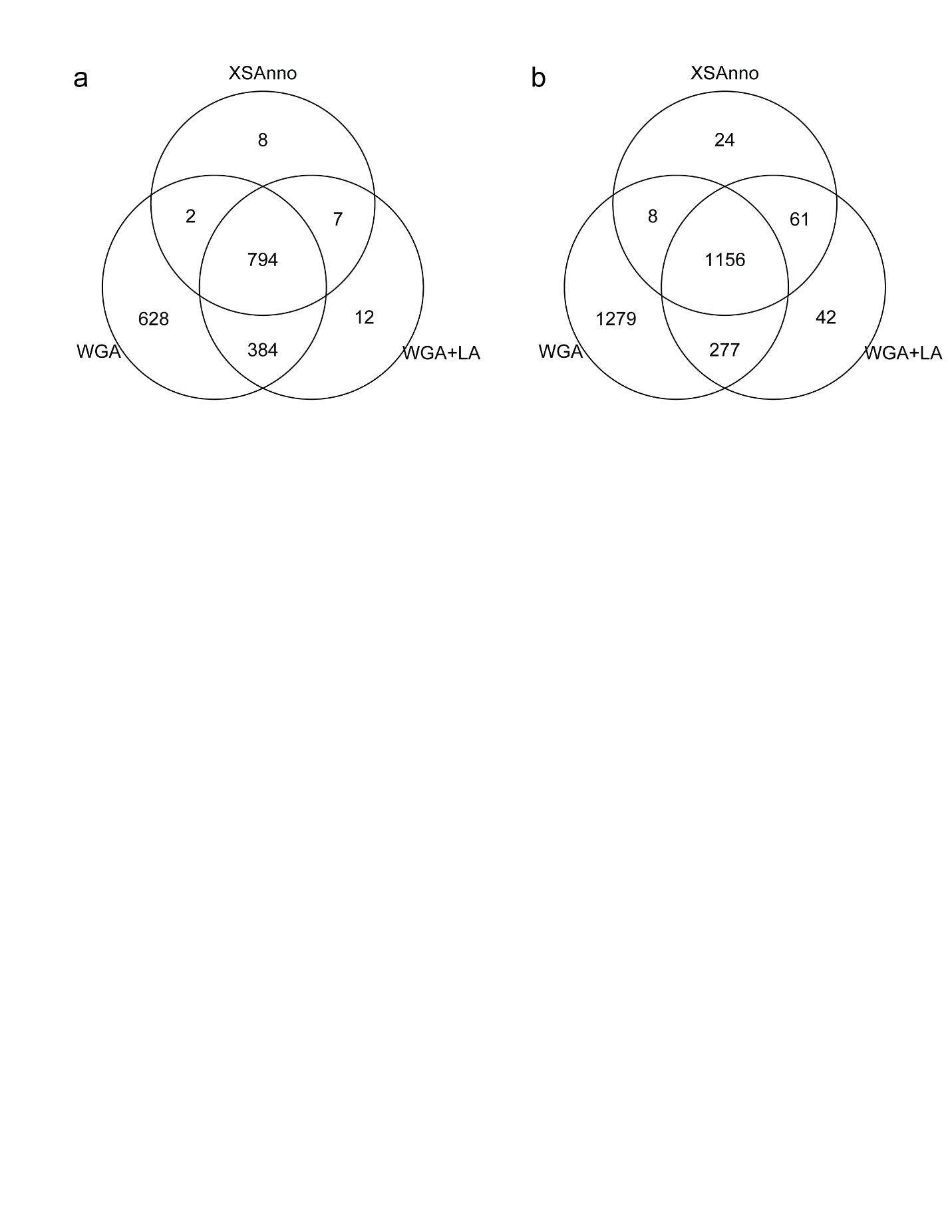
**

**Figure S9. The number of differentially expressed genes**

The number of differentially expressed (DEX) genes identified between human and chimpanzee (a) and between human and macaque (b), using annotations built in each step of XSAnno.
